# Diagnostic thresholds for pregnancy hyperglycemia, maternal weight status and the risk of childhood obesity in a diverse Northern California cohort using health care delivery system data

**DOI:** 10.1371/journal.pone.0216897

**Published:** 2019-05-10

**Authors:** Samantha F. Ehrlich, Monique M. Hedderson, Fei Xu, Assiamira Ferrara

**Affiliations:** 1 Division of Research, Kaiser Permanente Northern California, Oakland, CA, United States of America; 2 Department of Public Health, The University of Tennessee Knoxville, Knoxville, TN, United States of America; University at Buffalo, UNITED STATES

## Abstract

**Objective:**

To estimate the risk of childhood obesity associated with the various criteria proposed for diagnosis of gestational diabetes (GDM), and the joint effects with maternal BMI.

**Methods:**

Cohort study of 46,396 women delivering at the Kaiser Permanente Northern California health care delivery system in 1995–2004 and their offspring, followed through 5–7 years of age. Pregnancy hyperglycemia was categorized according to the screening and oral glucose tolerance test values proposed for the diagnosis of GDM by the International Association of the Diabetes and Pregnancy Study Group (IADPSG), Carpenter Coustan (CC), and the National Diabetes Data Group (NDDG). Childhood obesity was defined by the International Obesity Task Force’s age and sex-specific BMI cut-offs. Poisson regression models estimated the risks of childhood obesity associated with each category of pregnancy glycemia compared to normal screening, and the joint effects of maternal BMI category and GDM by the CC and the IADPSG criteria.

**Results:**

Compared with normal screening, increased risks of childhood obesity were observed for abnormal screening [RR (95% CI): 1.30 (1.22, 1.38)], 1+ abnormal values by the IADPSG or CC [1.47 (1.36, 1.59) and 1.48 (1.37, 1.59), respectively], and 2+ values by CC or NDDG [1.52 (1.39, 1.67) and 1.60 (1.43, 1.78), respectively]. Compared to obese women without GDM, obese women with GDM defined by the CC criteria had significantly increased risk of childhood obesity [1.20 (1.07, 1.34)], which was also observed for GDM by the IADSPG [1.18 (1.07, 1.30)], though GDM did not significantly increase the risk of childhood obesity among normal weight or overweight women.

**Conclusions:**

The risk of childhood obesity starts to increase at levels of pregnancy glycemia below those used to diagnose GDM and the effect of GDM on childhood obesity risk appears more pronounced in women with obesity. Interventions to reduce obesity and pregnancy hyperglycemia are warranted.

## Introduction

*In utero* exposure to maternal hyperglycemia increases the risk of childhood obesity [1]. Gestational diabetes (GDM), or diabetes first recognized during pregnancy, affects up to 9.2% of pregnancies in the U.S. [2]. Screening for GDM is nearly universal in most clinical settings since its treatments reduces the incidence of several perinatal complications [3, 4]. However, there is a lack of consensus regarding which of several proposed criteria for the identification GDM should be used in clinical practice and an on-going clinical debate on whether reducing maternal overweight and obesity or treating pregnancy hyperglycemia is the more salient public health strategy for preventing childhood obesity. In regard to the criteria for GDM diagnosis, the American Diabetes Association [5] currently recommends either the two-step approach (i.e., screening followed by a diagnostic test) with the Carpenter and Coustan (CC) criteria [6] or the one-step approach with the International Association of the Diabetes and Pregnancy Study Groups (IADPSG) criteria [7], which is recommended by the World Health Organization [8] and uses lower glycemic thresholds than the CC criteria. The American College of Obstetricians and Gynecologists [9] currently supports the two-step approach with either the CC criteria or the less frequently used National Diabetes Data Group (NDDG) criteria [10], which uses the highest glycemic thresholds of all. Although the associations of the CC and NDGG criteria with the risk of childhood obesity have been evaluated [11, 12], no studies have evaluated the risk of childhood obesity across all of the criteria currently used for the diagnosis of GDM. In addition, although maternal overweight and obesity is a well-documented risk factor for both GDM and childhood obesity [13], the degree to which the effect of maternal overweight and obesity on childhood obesity risk is compounded by the presence of pregnancy hyperglycemia remains largely unknown.

This study sought to fill these gaps in the literature by estimating the associations of the various pregnancy glucose criteria and thresholds recommended for the diagnosis of GDM with childhood obesity at 5 to 7 years of age, both overall and across racial-ethnic groups. The study additionally sought to investigate the joint effects of maternal BMI category and pregnancy hyperglycemia on subsequent childhood obesity risk.

## Materials and methods

This cohort study took place at Kaiser Permanente Northern California (KPNC), a large integrated health care delivery system with an expansive electronic health record (EHR) system, from 1995 and 2011. KPNC’s membership includes approximately 30% of the geographic area served and is representative of the surrounding population in regards to sociodemographic characteristics, except at the lower extremes of income and education [14].

The results of all blood glucose testing were obtained from the KPNC Gestational Diabetes and Pregnancy Glucose Tolerance Registry [15]; all plasma glucose measurements in this setting are performed using the hexokinase method at the KPNC regional laboratory. Women with recognized pre-gestational diabetes were identified in the KPNC Diabetes Registry [16] and excluded.

In this setting, the two-step approach is used to identify GDM: at 24–28 weeks gestation, women are screened with a 50-g, 1-hour glucose challenge test (> 95% of pregnancies are screened) and those with glucose ≥ 140 mg/dl (7.8 mmol/l) go on to a diagnostic 100-g, 3-hour oral glucose tolerance test (OGTT). During the study period (i.e., 1995–2004), women with ≥ 2 glucose values on the OGTT meeting or exceeding thresholds proposed by the National Diabetes Data Group [10] [NDDG, fasting: 105 mg/dl (5.8 mmol/l), 1-hour: 190 mg/dl (10.5 mmol/l), 2-hour: 165 mg/dl (9.1 mmol/l), and 3-hour: 145 mg/dl (8.0 mmol/l)] were diagnosed with GDM and received treatment. The lower thresholds proposed by Carpenter and Coustan [6] [CC, fasting: 95 mg/dl (5.3 mmol/l), 1-hour: 180 mg/dl (10.1 mmol/l), 2-hour: 155 mg/dl (8.7 mmol/l), and 3-hour: 140 mg/dl (7.8 mmol/l)] were implemented in this setting in 2007, thus women in the current study who met the CC criteria but not the NDDG criteria were not treated for hyperglycemia [17, 18].

The IADPSG [7] criteria were proposed in 2010 and utilize the one-step approach: a single 75-g, 2-hour OGTT with GDM diagnosed if any glucose value meets or exceeds the IADPSG thresholds [fasting: 92 mg/dl (5.1 mmol/l), 1-hour: 180 mg/dl (10.1 mmol/l), and 2-hour: 153 mg/dl (8.5 mmol/l)]. Although the one step procedure is not used in this setting, we assessed the IADPSG thresholds, which are based on a 75-g glucose load, and applied them to the 100-g OGTT to provide conservative estimates of the IADPSG with childhood obesity.

To examine associations with the *diagnostic criteria* for GDM, women were categorized into the following non-mutually exclusive categories: 1) abnormal screening; 2) abnormal screening and one or more 100 g, 3-hour OGTT values meeting the IADPSG thresholds for fasting, 1-hour or 2-hours; 3) abnormal screening and one or more 100 g, OGTT value meeting the CC thresholds for fasting, 1-hour, 2-hours or 3-hours; 4) abnormal screening and two or more 100 g, OGTT values meeting the CC thresholds for fasting, 1-hour, 2-hours or 3-hours; and 5) abnormal screening and two or more 100 g, OGTT values meeting the NDDG criteria for fasting, 1-hour, 2-hours or 3-hours. These categories, based on the various *diagnostic criteria* for GDM, were each examined in separate models and women with normal screening values (glucose <140 mg/dl) served as the reference.

To examine pregnancy hyperglycemia categorized by the *glucose thresholds* for the fasting, 1-hour and 2-hour time points of the 100-g, 3-h OGTT, women were divided into non-mutually exclusive groups for time point specific analyses (i.e., separate analyses conducted for fasting, 1-hour and 2-hour): 1) abnormal screening; 2) abnormal screening and glucose meeting or exceeding the IADPSG threshold for that time point; 3) abnormal screening and glucose meeting or exceeding the CC threshold for that time point; and 4) abnormal screening and glucose meeting or exceeding the NDDG threshold for that time point. Women with normal screening (<140 mg/dl) also served as the reference group.

The weights and heights of the children at 5 to 7 years of age were obtained from the EHR system; for children who had multiple measurements during this time frame, the measurements closest to 6 years of age were selected. BMI was calculated as weight (kilograms) divided by the height (meters) squared and the extended International Obesity Task Force’s (IOTF) BMI cut-offs [19] used to identify childhood obesity. Briefly, these age and sex specific cut-offs are based upon predicted adult BMI cut-offs for weight status [19, 20] As such, childhood obesity was defined by the age and sex specific cut-offs for a projected BMI ≥ 30 kg/m^2^ at 18 years of age. Sensitivity analyses utilized the Centers for Disease Control and Prevention’s (CDC) age and sex specific growth standards [21] to identify childhood obesity (i.e., ≥ 95^th^ percentile).

Maternal age at delivery, parity, height, early pregnancy weight, and gestational age at the pregnancy weight measurement, plus child’s sex and age at the weight and height measurement, were obtained from the EHR. The early pregnancy weight measurement occurred, on average, at 16.9 weeks gestation (SD 1.3) and maternal BMI was classified <18.5 kg/m^2^ (underweight), 18.5–24.9 kg/m^2^ (normal weight), 25.0–29.9 kg/m^2^ (overweight) or ≥30 kg/m^2^ (obese). Data on self-reported race-ethnicity and educational attainment were obtained via linkage with the state of California birth certificate (99% successful linkage [22]).

We identified 48,998 pregnant women, 18–45 years of age, who were screened for GDM and delivered a singleton infant in January 1995-December 2004, and whose child was a KPNC member at 5–7 years of age (i.e., through 2011). If a woman had more than one pregnancy in this period, the first was selected. There were 2,602 women excluded for missing data on early pregnancy weight. The final analytic cohort consisted of 46,396 mother-child pairs.

This study was approved by the Kaiser Foundation Research Institute and the state of California institutional review boards. The human subjects committee of the Kaiser Foundation Research Institute waived the requirement for individual informed consent. All analyses conducted in SAS.

### Statistical analyses

Poisson regression models with robust standard errors [23] were used to estimate the risks of childhood obesity associated with the categories of pregnancy glycemia. Two approaches were used to categorize and examine pregnancy glycemia. In the first, pregnancy glycemia was categorized into non-mutually exclusive groups based on the *diagnostic criteria* for GDM: abnormal screening, abnormal screening and 1+ abnormal OGTT values by the IADPSG thresholds (i.e., fasting, 1-hour or 2-hour values only), abnormal screening and 1+ abnormal OGTT value by the CC thresholds (i.e., fasting, 1-hour, 2-hour, or 3-hour values), abnormal screening and 2+ abnormal values by the CC criteria (i.e., fasting, 1-hour, 2-hour, or 3-hour values), and abnormal screening and 2+ abnormal values by the NDDG criteria (i.e., fasting, 1-hour, 2-hour, or 3-hour values). Separate models were constructed to compare each *diagnostic criteria* category to women with normal screening values.

For the second approach, non-mutually exclusive *glucose threshold* categories were determined by the time point specific thresholds of the IADPSG, CC and NDDG criteria. Time point specific analyses were then conducted: separate models were run for each *glucose threshold* category for the fasting, 1-hour and 2-hour time points of the OGTT, and women with normal screening values served as the reference in all models.

Interaction terms for pregnancy glucose, by both classification schemes, with maternal BMI and race-ethnicity were also examined.

The only Table 1 variable to alter risk ratio estimates by 10% or more was maternal BMI; maternal race-ethnicity and age were selected as additional adjustment variables. Multivariable models included maternal pregnancy glycemia, BMI (i.e., <18.5 kg/m^2^, 18.5–24.9 kg/m^2^, 25.0–29.9 kg/m^2^ and ≥30.0 kg/m^2^), race-ethnicity (i.e., White, Hispanic, Asian, African American, and Other) and age (continuous).

**Table 1:** Characteristics of the 46,396 women delivering at Kaiser Permanente Northern California in 1995–2004.

	Total	Without GDM	GDM
	N = 46,396	N = 43,476	N = 2,920
	n (%)	n (%)	n (%)
**Maternal age at delivery**, years			
18–24	10,381 (22.4)	10,122 (23.3)	259 (8.9)
25–29	15,161 (32.7)	14,378 (33.1)	783 (26.8)
30–34	15,336 (33.1)	14,116 (32.5)	1,220 (41.8)
35–45	5,581 (11.9)	4,860 (11.2)	658 (22.5)
**Maternal race-ethnicity**			
White	17,254 (37.2)	16,486 (37.9)	768 (26.3)
Hispanic	12,740 (27.5)	11,910 (27.4)	830 (28.4)
Asian	10,823 (23.3)	9,751 (22.4)	1,072 (36.7)
African American	4,051 (8.7)	3,883 (8.9)	168 (5.8)
Other	1,528 (3.3)	1,446 (3.3)	82 (2.8)
Maternal education*			
Some High School or High School graduate	15,955 (35.4)	14,990 (35.5)	965 (33.8)
Some college	13,424 (29.8)	12,570 (29.8)	854 (29.9)
College graduate or beyond	15,719 (34.9)	14,685 (34.8)	1,034 (36.2)
Parity*			
0	27,654 (59.6)	26,061 (60.0)	1,593 (54.6)
1	11,592 (25.0)	10,848 (25.0)	744 (25.5)
2+	7,134 (15.4)	6,551 (15.1)	583 (20.0)
**Maternal BMI Category,** kg/m^2^			
<18.5	853 (1.8)	829 (1.9)	24 (0.8)
18.5–24.9	21,273 (45.9)	20,371 (46.9)	902 (30.9)
25.0–29.9	14,211 (30.6)	13,293 (30.6)	918 (31.4)
≥30.0	10,059 (21.7)	8,983 (20.7)	1,076 (36.9)

* There were n = 1,298 women missing data on education and n = 16 missing data on parity

GDM: gestational diabetes, defined by either the Carpenter and Coustan or National Diabetes Data Group criteria, BMI: body mass index

The joint impact of maternal BMI and GDM was also examined, with GDM defined in two ways: (a) meeting the CC criteria for GDM (i.e., 2+ abnormal values by the CC), or (b) 1+ abnormal values by the IADPSG thresholds for fasting, 1-hour, or 2-hours or meeting the full CC criteria for GDM. Women with a BMI < 18.5 kg/m^2^ (underweight) were excluded from these analyses due to the small number of women with GDM (n = 24). A Poisson regression model was then constructed with the following categories: pregnancy BMI 18.5–24.9 kg/m^2^ (normal weight) and no GDM (reference); pregnancy BMI 18.5–24.9 kg/m^2^ and GDM; pregnancy BMI 25.0–29.9 kg/m^2^ (overweight) and no GDM; pregnancy BMI 25.0–29.9 kg/m^2^ and GDM; pregnancy BMI ≥30.0 kg/m^2^ (obese) and no GDM; and pregnancy BMI ≥30.0 kg/m^2^ and GDM. To arrive at estimates of the effects of GDM within the overweight and obese strata, the reference group was set to women with overweight and no GDM, and obesity and no GDM, in subsequent models.

## Results

Over half of the women had early pregnancy BMI ≥25.0 kg/m^2^ (Table 1). The children’s weight and height measurements at 5 to 7 years of age occurred, on average, at 6.3 years of age (SD = 0.7); 4,900 children (10.6%) were classified as obese according to the International Obesity Task Force’s cut-offs and 7,360 (15.9%) were obese according to the CDC growth standards.

In the unadjusted models evaluating pregnancy glycemia categorized into non-mutually exclusive groups based on the various *diagnostic criteria* for GDM, the risk of childhood obesity by the International Obesity Task Force’s cut-offs was increased for all categories of pregnancy hyperglycemia (Table 2). As compared to normal screening, the risk of childhood obesity was statistically significantly elevated among women with abnormal screening [RR = 1.30 (95% CI 1.22, 1.38)]; abnormal screening plus one or more abnormal OGTT values by the IADPSG thresholds [RR = 1.47 (95% CI 1.36, 1.59)]; abnormal screening plus one or more abnormal OGTT values by the CC thresholds [RR = 1.47 (95% CI 1.37, 1.59)]; abnormal screening plus two or more abnormal OGTT values by the CC criteria [RR = 1.52 (95% CI 1.39, 1.67)]; and abnormal screening plus two or more abnormal OGTT values by the NDDG criteria [RR = 1.60 (95% CI 1.43, 1.78); Table 2]. Following adjustment for maternal BMI, race-ethnicity, and age, all risk estimates were attenuated but remained statistically significant (Table 2). Risk estimates were attenuated but remained statistically significant with childhood obesity defined according to the CDC growth standards (S1 Table).

**Table 2:** Risk Ratio estimates and 95% confidence intervals for associations of the Diagnostic Criteria for gestational diabetes with childhood obesity at 5–7 years of age among 46,396 women delivering at Kaiser Permanente Northern California in 1995–2004.

		Childhood Obesity		
			Unadjusted	Adjusted^*^
	N women	n cases of childhood obesity	RR (95% CI)	RR^*^ (95% CI)
**Diagnostic Criteria for GDM**				
Normal screening	38,184	3,830	Reference	Reference
Abnormal screening	8,212	1,070	1.30 (1.22, 1.38)	1.13 (1.06, 1.20)
Abnormal screening and 1+ abnormal OGTT values by IADPSG	4,431	654	1.47 (1.36, 1.59)	1.18 (1.09, 1.27)
Abnormal screening and 1+ abnormal OGTT value by CC	4,392	650	1.48 (1.37, 1.59)	1.19 (1.10, 1.29)
Abnormal screening and 2+ abnormal OGTT values by CC	2,731	417	1.52 (1.39, 1.67)	1.20 (1.09, 1.32)
Abnormal screening and 2+ abnormal OGTT values by NDDG	1,825	292	1.60 (1.43, 1.78)	1.25 (1.12, 1.39)

^*^ Adjusted for maternal age, race-ethnicity, and BMI category

OGTT: 100g, 3-hr oral glucose tolerance test, IADPSG: International Association of Diabetes in Pregnancy Study Groups, CC: Carpenter and Coustan, NDDG: National Diabetes Data Group

Note that the diagnostic criteria categories are not mutually exclusive, RR estimates obtained from separate models

Table 3 shows the risk estimates for pregnancy hyperglycemia categorized into non-mutually exclusive groups based on the *glucose thresholds* for the fasting, 1-hour and 2-hour time points of the 100-g OGTT. In the unadjusted models, the risk of childhood obesity by the International Obesity Task Force’s cut-offs was significantly increased for women with abnormal screening as compared to normal screening, as well as for all higher degrees of pregnancy hyperglycemia at each time point. For the fasting, 1-hour and 2-hour time points, women with abnormal screening who additionally met any of the time point specific glucose thresholds had significantly increased risks for childhood obesity (RR ranges: 1.86–2.68 for fasting, 1.57–1.61 for 1-hour, and 1.42–1.47 for 2-hour).

**Table 3:** Risk Ratio estimates and 95% confidence intervals for the associations of the fasting, 1-hour and 2-hour Glucose Threshold Categories with childhood obesity at 5–7 years of age among 46,396 women delivering at Kaiser Permanente Northern California in 1995–2004.

		Childhood Obesity		
			Unadjusted	Adjusted^*^
	N women	n cases of childhood obesity	RR (95% CI)	RR^*^ (95% CI)
**Fasting Glucose Thresholds**				
Normal screening	38,184	3,830	Reference	Reference
Abnormal screening	8,212	1,070	1.30 (1.22, 1.38)	1.13 (1.06, 1.20)
Abnormal screening and fasting glucose ≥92 mg/dl^†^	1,751	334	1.86 (1.69, 2.06)	1.27 (1.15, 1.40)
Abnormal screening and fasting glucose ≥95 mg/dl^‡^	1,277	259	2.02 (1.81, 2.26)	1.32 (1.18, 1.48)
Abnormal screening and fasting glucose ≥105 mg/dl^§^	439	118	2.68 (2.29, 3.14)	1.59 (1.35, 1.86)
**1-hour Glucose Thresholds**				
Normal screening	38,184	3,830	Reference	Reference
Abnormal screening	8,212	1,070	1.30 (1.22, 1.38)	1.13 (1.06, 1.20)
Abnormal screening, 1-hour glucose ≥180 mg/dl^¶^	3,044	478	1.57 (1.43, 1.71)	1.23 (1.13, 1.35)
Abnormal screening, 1-hour glucose ≥190 mg/dl^§^	2,183	353	1.61 (1.46, 1.78)	1.24 (1.12, 1.37)
**2-hour Glucose Thresholds**				
Normal screening	38,184	3,830	Reference	Reference
Abnormal screening	8,212	1,070	1.30 (1.22, 1.39)	1.13 (1.06, 1.20)
Abnormal screening, 2-hour glucose ≥153 mg/dl^†^	3,290	478	1.42 (1.30, 1.55)	1.17 (1.07, 1.27)
Abnormal screening, 2-hour glucose ≥155 mg/dl^‡^	3,134	462	1.47 (1.34, 1.61)	1.20 (1.09, 1.31)
Abnormal screening, 2-hour glucose ≥165 mg/dl^§^	2,239	325	1.45 (1.30, 1.61)	1.18 (1.06, 1.30)

^*^ Adjusted for maternal age, race-ethnicity, and BMI category

^†^ Meeting the International Association of Diabetes in Pregnancy Study Groups threshold

^‡^ Meeting the Carpenter and Coustan threshold

^§^ Meeting National Diabetes Data Group threshold

^¶^ Meeting the International Association of Diabetes in Pregnancy Study Groups/Carpenter and Coustan thresholds, which are identical for the 1-hour time point

IADPSG: International Association of Diabetes in Pregnancy Study Groups, CC: Carpenter and Coustan, NDDG: National Diabetes Data Group

Note that the glucose threshold categories are not mutually exclusive, RR estimates obtained from separate models

Following adjustment for maternal BMI, race-ethnicity, and age, the risk estimates for the *glucose threshold* categories were all attenuated but remained statistically significant (Table 3). The adjusted risk estimates for the association of fasting glycemia with childhood obesity were 1.27 (95% CI 1.15, 1.40) for those meeting or exceeding the IADPSG threshold (i.e., fasting glucose ≥92 mg/dl); 1.32 (95% CI 1.18, 1.48) for those meeting or exceeding the CC threshold (i.e., fasting glucose ≥95 mg/dl); and 1.59 (95% CI 1.35, 1.86) for those meeting or exceeding the NDDG threshold (i.e., fasting glucose ≥105 mg/dl) compared to normal screening. For 1-hour glycemia, the adjusted risk of childhood obesity remained significantly elevated for those who met the CC/IADPSG threshold for 1-hour glucose [i.e., 1-hour glucose ≥180 mg/dl, RR = 1.23 (95% CI 1.13, 1.35)] or those who met or exceeded the NDDG threshold [i.e., 1-hour glucose ≥190 mg/dl: RR = 1.24 (95% CI 1.12, 1.37)]. For 2-hour glycemia, the adjusted risk of childhood obesity remained significantly elevated among women who met the IADPSG threshold (i.e., 2-hour glucose ≥153 mg/dl; RR = 1.17 (95% CI 1.07, 1.27); the CC threshold [i.e., 2-hour glucose ≥155 mg/dl: RR = 1.20 (95% CI 1.09, 1.31)] or who met or exceeded the NDDG threshold [i.e., 2-hour glucose ≥165 mg/dl: RR = 1.18 (95% CI 1.06, 1.30)]. With childhood obesity defined according to the CDC growth standards, risk estimates were attenuated but remained statistically significant (S2 Table).

The risk of childhood obesity by the International Obesity Task Force’s cut-offs was also increased among women with overweight or obesity, and among African American and Hispanic women. In a crude model with just maternal BMI, the risk of childhood obesity was RR = 2.36 (95% CI 2.19, 2.54) for maternal BMI 25.0–29.9 kg/m^2^ and RR = 4.44 (95% CI 4.14, 4.76) for maternal BMI ≥ 30 kg/m^2^ compared to BMI 18.5–24.9 kg/m^2^; for maternal BMI < 18.5 kg/m^2^, the risk was RR = 0.43 (95% CI 0.27, 0.68). In a crude model with just maternal race-ethnicity, the risk was RR = 1.92 (95% CI 1.76, 2.11) for African American women, RR = 2.08 (95% CI 1.95, 2.22) for Hispanic women, and RR = 0.99 (95% CI 0.91, 1.08) for Asian women compared to White women. Interaction terms for pregnancy glucose with maternal BMI and race-ethnicity were added to the models presented in Tables 2 and 3. The interaction term for abnormal screening and maternal BMI attained statistical significance (*P* < .10 for BMI categorical and *P* < .01 for BMI continuous). In analyses stratified by maternal BMI (underweight and normal weight versus overweight and obese, presented in S3 and S4 Tables), similar findings were observed, though several adjusted risks estimates did not attain statistical significance among the underweight and normal weight women. The interaction between abnormal screening and race-ethnicity also attained statistical significance (*P* < .05). In analyses stratified by maternal race-ethnicity, similar findings were observed for each racial-ethnic group (S5, S6, S7 and S8 Tables).

**Fig 1:**
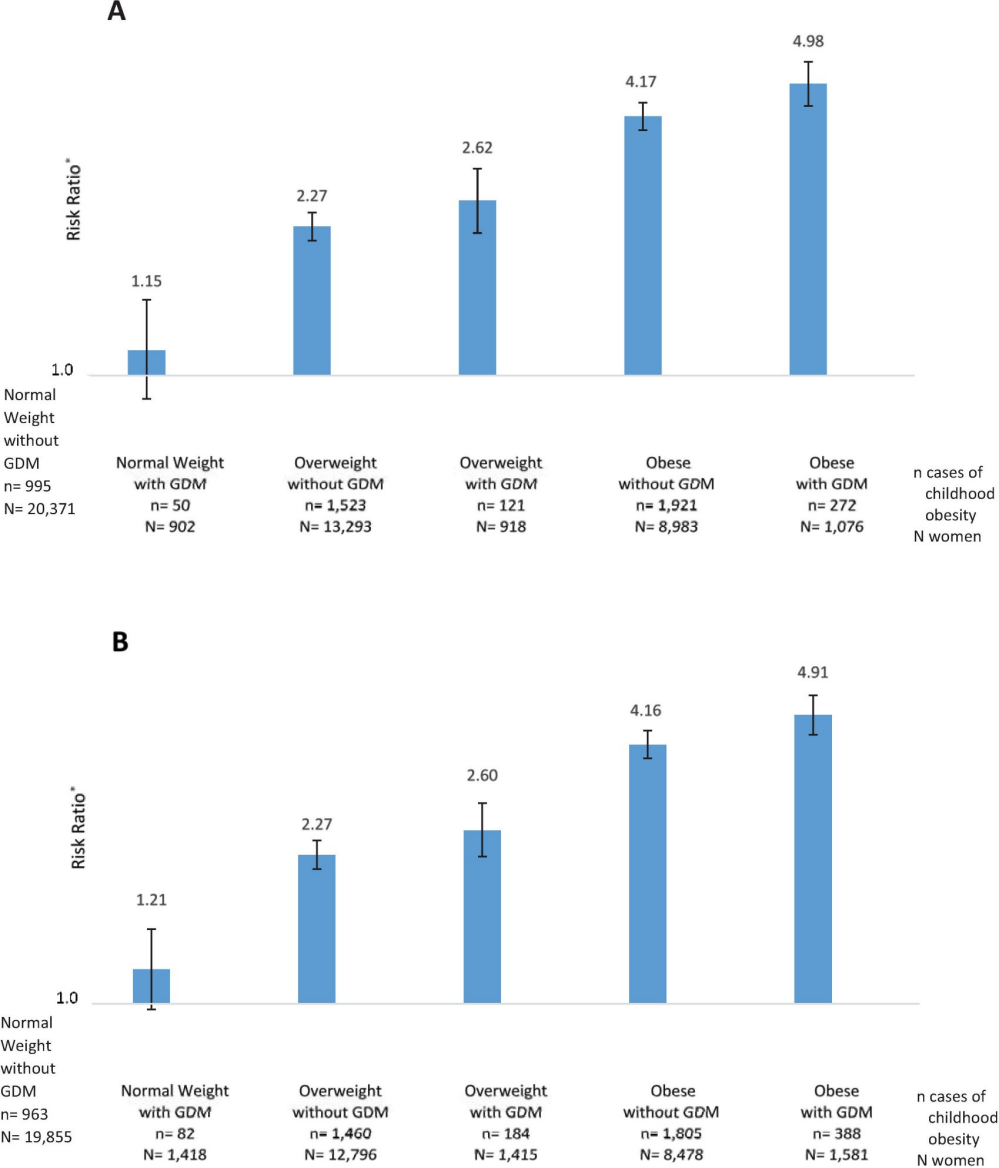
Adjusted^*^ risk ratio estimates and 95% confidence intervals for the associations of the joint maternal BMI and GDM status categories with childhood obesity at 5–7 years of age among 45,543 women delivering at Kaiser Permanente Northern California in 1995–2004. ^*^ Among n = 45,543 with BMI ≥ 18.5 kg/m^2^; Adjusted for maternal age and race-ethnicity; women classified as normal weight (BMI 18.5–24.9 kg/m^2^) without GDM serve as the reference. Panel A: GDM defined by the CC criteria, n = 20,371 normal weight without GDM as the reference. Panel B: GDM definition expanded to include meeting one or more of the IADPSG thresholds for the fasting, 1-hr or 2-hr time points of the 100-g, 3-h OGTT or the CC criteria, n = 19,855 normal weight without GDM as the reference. BMI: body mass index, GDM: gestational diabetes, CC: Carpenter and Coustan, NDDG: National Diabetes Data Group, IADPSG: International Association of Diabetes in Pregnancy Study Groups.

Fig 1 displays the risk estimates for the joint effects of maternal BMI category and GDM status defined by the CC criteria (i.e. 2+ CC abnormal values, Panel A) or expanded to include the IADPSG criteria (i.e. 1+ IADPSG abnormal value by the fasting, 1-hour, or 2-hour time points or 2+ CC abnormal values, Panel B). When GDM was defined by the CC criteria alone (n = 2,896 with GDM by the CC criteria alone, Panel A), as compared to women with normal weight and without GDM, those classified as overweight without GDM had an increased risk of childhood obesity [RR = 2.27 (95% CI 2.10, 2.45)] and the risk increased further for those classified as overweight with GDM [RR = 2.62 (95% CI 2.19, 3.12)], as obese without GDM [RR = 4.17 (95% CI 3.86, 4.49)] and as obese with GDM [RR = 4.98 (95% CI 4.41, 5.62); Fig 1]. The risk of childhood obesity in women with normal weight or overweight and GDM did not differ from that observed among women in these same weight categories without GDM [RR = 1.15 (95% CI 0.88, 1.52) for normal weight, Fig 1, and RR = 1.15 (95% CI 0.97, 1.37) for overweight, from a separate model]. However, as compared to women with obesity who did not have GDM, those with obesity and GDM had a significantly increased risk of childhood obesity [RR = 1.20 (95% CI 1.07, 1.34), from a separate model].

When the definition of GDM was expanded to include 1+ abnormal value by the IADPSG thresholds for the fasting, 1-hour or 2-hour time points (n = 4,414 with GDM by the CC criteria or meeting 1+ IADPSG thresholds, Panel B, Fig 1), similar risk estimates were obtained for each maternal BMI and GDM status subgroup, and the impact of GDM on childhood obesity risk within strata of maternal weight status was unaltered. The risk of childhood obesity in women with normal or overweight did not differ from that observed among women in the same weight category without GDM [RR = 1.21 (95% CI 0.97, 1.51) for normal weight, Fig 1, and RR = 1.15 (95% CI 0.99, 1.32) for overweight, from a separate model], though women with obesity and GDM had significantly increased risk of childhood obesity as compared to women with obesity alone [RR = 1.18 (95% CI 1.07, 1.30) for obese, from a separate model].

## Discussion

The results of this study suggest that the risk of childhood obesity starts to increase with pregnancy hyperglycemia detected by an abnormal screening value alone. The risk among women with a single abnormal value by the IADPSG thresholds was nearly the same as that observed among women with a single abnormal value by the CC thresholds and similar to risk observed among women with two or more abnormal values by the CC criteria, though only the later are typically diagnosed and treated for GDM. The risk of childhood obesity was further increased among women who met the full NDDG criteria, the only group to receive treatment for GDM in this setting during the study period. These findings suggest that in a clinical setting, an abnormal screening test and/or a single abnormal value by either the IADPSG or the CC thresholds could be used to identify infants at increased risk of childhood obesity. However, it should be noted that the prevalence of GDM would increase by at least 60.6% (22) if a single abnormal value by either the CC or the IADPSG thresholds were used to diagnose GDM. Therefore, the benefits of treating women meeting these lower thresholds must be considered alongside the increased burden on the health care system and cost of treatment.

Following adjustment for maternal age, race-ethnicity and BMI, the risk estimates for childhood obesity were attenuated but remained statistically significant, suggesting that, etiologically, pregnancy hyperglycemia increases the risk of childhood obesity independently of recognized maternal risk factors. Importantly, similar associations between the pregnancy glucose thresholds and criteria and risk of childhood obesity were observed across all racial-ethnic groups. The risk of childhood obesity increased with increasing maternal BMI and the presence of GDM (whether defined by the CC or IASPDG criteria) further increased the risk among women with obesity. Although there were small numbers of women with GDM among the normal or overweight women, the risk of childhood obesity in those with normal weight or overweight was not significantly increased by the presence of GDM. Taken together, the results of this study suggest that maternal BMI may be the more salient, modifiable risk factor for childhood obesity than GDM.

Consistent with the results of the current study, a follow-up study to the Hyperglycemia and Adverse Pregnancy Outcome (HAPO) study [from which the IADPSG thresholds originated [24]], conducted at 10 of the 15 original HAPO field centers recently reported that the frequency of childhood obesity at 10 to 14 years of age progressively increased across mothers without GDM, those with IADPSG-defined GDM (i.e., meeting the IADPSG but not the CC), and those with CC-defined GDM (i.e., 2 to 3 glucose values at or above the CC thresholds applied to the 75-g OGTT), and that the trend attained statistical significance [25]. As in the current study, GDM defined by the IADPSG criteria was statistically significantly associated with childhood obesity following adjustment for pregnancy BMI [25]. A follow-up study conducted at a single HAPO field center, the Belfast Centre [26], found that fasting pregnancy hyperglycemia defined by the IADPSG threshold was associated with increased risk of childhood obesity in a sample of 1,320 children, 5 to 7 years of age, but the association was no longer significant following adjustment for pregnancy BMI [26]. A follow-up study of 970 ethnic Chinese women conducted at the Hong Kong HAPO field center reported that GDM defined by the IADPSG criteria was significantly associated with offspring overweight or obesity at 7 years of age and that the results remained significant following adjustment for prepregnancy BMI [27]. Yet a large population-based cohort study in Tianjin, China of 27,155 mother-child pairs found that GDM defined by the IADPSG criteria was significantly associated with a higher mean BMI for age *Z*-score through 6 years of age; the difference remained statistically significant at 5 years of age but disappeared for 4 and 6 years of age with adjustment for prepregnancy BMI [28]. These inconsistent findings may be explained by a number of factors, including sample size, population differences (e.g., genetic susceptibility) and/or the use of different methods to assess pregnancy hyperglycemia, alternately, they could reflect true variation of effects over the course of childhood.

Although treatment for GDM bestows benefits in terms of neonatal outcomes (e.g., infant birth weight, macrosomia, and neonatal fat mass [3, 4]), the evidence pertaining to childhood obesity is less clear. The *Eunice Kennedy Shriver* National Institute of Child Health and Human Development Maternal-Fetal Medicine Units (MFMU) Network randomized clinical trial assessing the effects of treatment on perinatal adverse outcomes among women with mild GDM (i.e., fasting glucose <95 mg/dl and two of three timed measurements exceeding the CC thresholds) conducted a follow-study and found no difference in obesity at 5 to 10 years of age by treatment status [29]. In the current study, those who met the NDDG criteria for GDM and received treatment similarly did not show a reduced risk of childhood obesity. However, in a large observational study, Hillier et al. [11] found that women with GDM by the CC criteria who were not treated had an increased odds of childhood obesity at 5 to 7 years of age, but those with GDM by the NDDG criteria who received treatment were not at statistically significant increased risk [i.e., OR = 1.38 (95% CI 0.84, 2.27)]. Additional studies are needed to assess whether treatment of GDM may reduce the risk of childhood obesity.

The current study was conducted in a large, diverse cohort of pregnant women representing the complete range pregnancy glycemia, a clear strength. A limitation of the current study is the lack of data on gestational weight gain, which is associated with both GDM [30] and childhood obesity [31] thus may confound the association of interest. We also assessed the IADPSG thresholds with a two-step procedure and a 100-g OGTT instead of a 75-g OGTT, as intended [7], thereby providing conservative estimates of the IADPSG glucose thresholds’ associations with childhood obesity due to the use of a higher glucose load and the fact that some of the women with normal screening (our reference group) may have had fasting, 1-hour or 2-hour glucose values meeting the IADPSG thresholds but could not be captured since they received only the screening test.

The results of this study suggest that the risk of childhood obesity is present at pregnancy glycemia levels below those recommended for the diagnosis of GDM and it increases with increasing level of pregnancy glycemia. The association between pregnancy hyperglycemia and childhood obesity is in large part explained by maternal overweight and obesity status. Examination of the joint impact of maternal BMI and GDM revealed that GDM may only compound the risk of childhood obesity in women with obesity. In terms of the upstream prevention of childhood obesity, interventions that identify women at risk of GDM early in pregnancy and aim to reduce pregnancy hyperglycemia as well as interventions that aim to at reduce obesity among reproductive aged women are warranted.

## Supporting information

S1 Table Risk ratio estimates and 95% Confidence Intervals for associations of the Diagnostic Criteria for gestational diabetes with childhood obesity at 5–7 years of age, identified by the Centers for Disease Control and Prevention’s growth standards, among 46,396 women delivering at Kaiser Permanente Northern California in 1995–2004. ^*^ Adjusted for maternal age, race-ethnicity, and BMI category. OGTT: 100g, 3-hr oral glucose tolerance test, IADPSG: International Association of Diabetes in Pregnancy Study Groups, CC: Carpenter and Coustan, NDDG: National Diabetes Data Group. Note that the diagnostic criteria categories are not mutually exclusive, RR estimates obtained from separate models.
(DOCX)

S2 Table Risk ratio estimates and 95% Confidence Intervals for the associations of the Fasting, 1-hour and 2-hour Glucose Threshold Categories with childhood obesity at 5–7 years of age, identified by the Centers for Disease Control and Prevention’s growth standards, among 46,396 women delivering at Kaiser Permanente Northern California in 1995–2004. ^*^ Adjusted for maternal age, race-ethnicity, and BMI category. ^†^ Meeting the International Association of Diabetes in Pregnancy Study Groups threshold. ^‡^ Meeting the Carpenter and Coustan threshold. ^§^ Meeting National Diabetes Data Group threshold ^¶^ Meeting the International Association of Diabetes in Pregnancy Study Groups/Carpenter and Coustan thresholds, which are identical for the 1-hour time point. IADPSG: International Association of Diabetes in Pregnancy Study Groups, CC: Carpenter and Coustan, NDDG: National Diabetes Data Group. Note that the glucose threshold categories are not mutually exclusive, RR estimates obtained from separate models.
(DOCX)

S3 Table Risk ratio estimates and 95% Confidence Intervals for the associations of the GDM Diagnostic Criteria and Glucose Threshold Categories with childhood obesity at 5–7 years of age, identified by International Obesity Task Force’s cut-offs, among underweight and normal weight women (n = 22,126), Kaiser Permanente Northern California, 1995–2011. ^*^ Multivariable models include the respective pregnancy glycemia variable, maternal age and BMI category (<18.5 kg/m^2^ and 18.5–24.9 kg/m^2^). ^†^ Meeting the International Association of Diabetes in Pregnancy Study Groups threshold. ^‡^ Meeting the Carpenter and Coustan threshold. ^§^ Meeting National Diabetes Data Group threshold. ^¶^ Meeting the International Association of Diabetes in Pregnancy Study Groups/Carpenter and Coustan thresholds, which are identical for the 1-hour time point. OGTT: 100g, 3-hr oral glucose tolerance test, IADPSG: International Association of Diabetes in Pregnancy Study Groups, CC: Carpenter and Coustan, NDDG: National Diabetes Data Group, CC: Carpenter and Coustan, NDDG: National Diabetes Data Group, BMI: body mass index Note that glucose categories are not mutually exclusive, RR estimates obtained from separate models.
(DOCX)

S4 Table Risk ratio estimates and 95% Confidence Intervals for the associations of the GDM Diagnostic Criteria and Glucose Threshold Categories with childhood obesity at 5–7 years of age, identified by International Obesity Task Force’s cut-offs, among women with overweight or obesity (n = 24,270), Kaiser Permanente Northern California, 1995–2011. ^*^ Multivariable models include the respective pregnancy glycemia variable, maternal age and BMI category (25.0–29.9 kg/m^2^ and ≥30.0 kg/m^2^) ^†^ Meeting the International Association of Diabetes in Pregnancy Study Groups threshold. ^‡^ Meeting the Carpenter and Coustan threshold. ^§^ Meeting National Diabetes Data Group threshold. ^¶^ Meeting the International Association of Diabetes in Pregnancy Study Groups/Carpenter and Coustan thresholds, which are identical for the 1-hour time point. OGTT: 100g, 3-hr oral glucose tolerance test, IADPSG: International Association of Diabetes in Pregnancy Study Groups, CC: Carpenter and Coustan, NDDG: National Diabetes Data Group, CC: Carpenter and Coustan, NDDG: National Diabetes Data Group, BMI: body mass index. Note that glucose categories are not mutually exclusive, RR estimates obtained from separate models.
(DOCX)

S5 Table Risk ratio estimates and 95% Confidence Intervals for the associations of the GDM Diagnostic Criteria and Glucose Threshold Categories with childhood obesity at 5–7 years of age, identified by International Obesity Task Force’s cut-offs, among White women (n = 17,254), Kaiser Permanente Northern California, 1995–2011. ^*^ Multivariable models include the respective pregnancy glycemia variable, maternal age and BMI category (<18.5 kg/m^2^, 18.5–24.9 kg/m^2^, 25.0–29.9 kg/m^2^, and ≥30.0 kg/m^2^). ^†^ Meeting the International Association of Diabetes in Pregnancy Study Groups threshold ^‡^ Meeting the Carpenter and Coustan threshold. ^§^ Meeting National Diabetes Data Group threshold. ^¶^ Meeting the International Association of Diabetes in Pregnancy Study Groups/Carpenter and Coustan thresholds, which are identical for the 1-hour time point. OGTT: 100g, 3-hr oral glucose tolerance test, IADPSG: International Association of Diabetes in Pregnancy Study Groups, CC: Carpenter and Coustan, NDDG: National Diabetes Data Group, CC: Carpenter and Coustan, NDDG: National Diabetes Data Group, BMI: body mass index. Note that glucose categories are not mutually exclusive, RR estimates obtained from separate models.
(DOCX)

S6 Table Risk ratio estimates and 95% Confidence Intervals for the associations of the GDM Diagnostic Criteria and Glucose Threshold Categories with childhood obesity at 5–7 years of age, identified by International Obesity Task Force’s cut-offs, among Hispanic women (n = 12,740), Kaiser Permanente Northern California, 1995–2011. ^*^ Multivariable models include the respective pregnancy glycemia variable, maternal age and BMI category (<24.9 kg/m^2^, 25.0–29.9 kg/m^2^, and ≥30.0 kg/m^2^). ^†^ Meeting the International Association of Diabetes in Pregnancy Study Groups threshold. ^‡^ Meeting the Carpenter and Coustan threshold. ^§^ Meeting National Diabetes Data Group threshold ^¶^ Meeting the International Association of Diabetes in Pregnancy Study Groups/Carpenter and Coustan thresholds, which are identical for the 1-hour time point. OGTT: 100g, 3-hr oral glucose tolerance test, IADPSG: International Association of Diabetes in Pregnancy Study Groups, CC: Carpenter and Coustan, NDDG: National Diabetes Data Group, CC: Carpenter and Coustan, NDDG: National Diabetes Data Group, BMI: body mass index. Note that glucose categories are not mutually exclusive, RR estimates obtained from separate models.
(DOCX)

S7 Table Risk ratio estimates and 95% Confidence Intervals for the associations of the GDM Diagnostic Criteria and Glucose Threshold Categories with childhood obesity at 5–7 years of age, identified by International Obesity Task Force’s cut-offs, among Asian women (n = 10,823), Kaiser Permanente Northern California, 1995–2011. ^*^ Multivariable models include the respective pregnancy glycemia variable, maternal age and BMI category (<18.5 kg/m^2^, 18.5–24.9 kg/m^2^, 25.0–29.9 kg/m^2^, and ≥30.0 kg/m^2^). ^†^ Meeting the International Association of Diabetes in Pregnancy Study Groups threshold ^‡^ Meeting the Carpenter and Coustan threshold. ^§^ Meeting National Diabetes Data Group threshold. ^¶^ Meeting the International Association of Diabetes in Pregnancy Study Groups/Carpenter and Coustan thresholds, which are identical for the 1-hour time point. OGTT: 100g, 3-hr oral glucose tolerance test, IADPSG: International Association of Diabetes in Pregnancy Study Groups, CC: Carpenter and Coustan, NDDG: National Diabetes Data Group, CC: Carpenter and Coustan, NDDG: National Diabetes Data Group, BMI: body mass index. Note that glucose categories are not mutually exclusive, RR estimates obtained from separate models.
(DOCX)

S8 Table Risk ratio estimates and 95% Confidence Intervals for the associations of the GDM Diagnostic Criteria and Glucose Threshold Categories with childhood obesity at 5–7 years of age, identified by International Obesity Task Force’s cut-offs, among African American women (n = 4,051), Kaiser Permanente Northern California, 1995–2011. ^*^ Multivariable models include the respective pregnancy glycemia variable, maternal age and BMI category (<18.5 kg/m^2^, 18.5–24.9 kg/m^2^, 25.0–29.9 kg/m^2^, and ≥30.0 kg/m^2^). ^†^ Meeting the International Association of Diabetes in Pregnancy Study Groups threshold. ^‡^ Meeting the Carpenter and Coustan threshold. ^§^ Meeting National Diabetes Data Group threshold. ^¶^ Meeting the International Association of Diabetes in Pregnancy Study Groups/Carpenter and Coustan thresholds, which are identical for the 1-hour time point. OGTT: 100g, 3-hr oral glucose tolerance test, IADPSG: International Association of Diabetes in Pregnancy Study Groups, CC: Carpenter and Coustan, NDDG: National Diabetes Data Group, CC: Carpenter and Coustan, NDDG: National Diabetes Data Group, BMI: body mass index. Note that glucose categories are not mutually exclusive, RR estimates obtained from separate models.
(DOCX)

## References

[1] Dabelea D, Harrod CS. Role of developmental overnutrition in pediatric obesity and type 2 diabetes. Nutr Rev. 2013;71 Suppl 1:S62–7. 24147926 10.1111/nure.12061

[2] DeSisto CL, Kim SY, Sharma AJ. Prevalence estimates of gestational diabetes mellitus in the United States, Pregnancy Risk Assessment Monitoring System (PRAMS), 2007–2010. Prev Chronic Dis. 2014;11:E104 10.5888/pcd11.130415 24945238 PMC4068111

[3] Crowther CA, Hiller JE, Moss JR, McPhee AJ, Jeffries WS, Robinson JS. Effect of treatment of gestational diabetes mellitus on pregnancy outcomes. N Engl J Med. 2005;352(24):2477–86. 10.1056/NEJMoa042973 15951574

[4] Landon MB, Spong CY, Thom E, Carpenter MW, Ramin SM, Casey B, et al A multicenter, randomized trial of treatment for mild gestational diabetes. N Engl J Med. 2009;361(14):1339–48. 10.1056/NEJMoa0902430 19797280 PMC2804874

[5] Classification and Diagnosis of Diabetes. Diabetes Care. 2017;40(Suppl 1):S11–s24. 10.2337/dc17-S005 27979889

[6] Carpenter MW, Coustan DR. Criteria for screening tests for gestational diabetes. Am J Obstet Gynecol. 1982;144(7):768–73. 7148898 10.1016/0002-9378(82)90349-0

[7] Metzger BE, Gabbe SG, Persson B, Buchanan TA, Catalano PA, Damm P, et al International association of diabetes and pregnancy study groups recommendations on the diagnosis and classification of hyperglycemia in pregnancy. Diabetes Care. 2010;33(3):676–82. 10.2337/dc09-1848 20190296 PMC2827530

[8] WHO Guidelines Approved by the Guidelines Review Committee. Diagnostic Criteria and Classification of Hyperglycaemia First Detected in Pregnancy. Geneva: World Health Organization Copyright (c) World Health Organization 2013. 24199271

[9] Practice Bulletin No. 180: Gestational Diabetes Mellitus. Obstet Gynecol. 2017;130(1):e17–e37. 10.1097/AOG.0000000000002159 28644336

[10] Classification and diagnosis of diabetes mellitus and other categories of glucose intolerance. National Diabetes Data Group. Diabetes. 1979;28(12):1039–57. 510803 10.2337/diab.28.12.1039

[11] Hillier TA, Pedula KL, Schmidt MM, Mullen JA, Charles MA, Pettitt DJ. Childhood obesity and metabolic imprinting: the ongoing effects of maternal hyperglycemia. Diabetes Care. 2007;30(9):2287–92. 10.2337/dc06-2361 17519427

[12] Hillier TA, Pedula KL, Vesco KK, Oshiro CE, Ogasawara KK. Impact of Maternal Glucose and Gestational Weight Gain on Child Obesity over the First Decade of Life in Normal Birth Weight Infants. Maternal and Child Health Journal. 2016. 10.1007/s10995-016-1955-7 PMC9870031 27154523

[13] Catalano PM, Shankar K. Obesity and pregnancy: mechanisms of short term and long term adverse consequences for mother and child. BMJ. 2017;356:j1 10.1136/bmj.j1 28179267 PMC6888512

[14] Gordon NP. Similarity of the Adult Kaiser Permanente Membership in Northern California to the Insured and General Population in Northern California: Statistics from the 2011–12 California Health Interview Survey Oakland, CA: Divisionof Research; 2015 [Available from: http://www.dor.kaiser.org/external/chis_non_kp_2011/

[15] Ferrara A, Kahn HS, Quesenberry CP, Riley C, Hedderson MM. An increase in the incidence of gestational diabetes mellitus: Northern California, 1991–2000. Obstet Gynecol. 2004;103(3):526–33. 10.1097/01.AOG.0000113623.18286.20 14990417

[16] Selby JV, Ray GT, Zhang D, Colby CJ. Excess costs of medical care for patients with diabetes in a managed care population. Diabetes Care. 1997;20(9):1396–402. 9283786 10.2337/diacare.20.9.1396

[17] Metzger BE, Coustan DR. Summary and recommendations of the Fourth International Workshop-Conference on Gestational Diabetes Mellitus. The Organizing Committee. Diabetes Care. 1998;21 Suppl 2:B161–7. 9704245

[18] Gestational diabetes mellitus. Diabetes Care. 2000;23 Suppl 1:S77–9. 12017686

[19] Cole TJ, Lobstein T. Extended international (IOTF) body mass index cut-offs for thinness, overweight and obesity. Pediatr Obes. 2012;7(4):284–94. 10.1111/j.2047-6310.2012.00064.x 22715120

[20] Cole TJ, Bellizzi MC, Flegal KM, Dietz WH. Establishing a standard definition for child overweight and obesity worldwide: international survey. BMJ. 2000;320(7244):1240–3. 10.1136/bmj.320.7244.1240 10797032 PMC27365

[21] CDC growth charts. Hyattsville, MD: National Center for Health Statistics; 2005.

[22] Ehrlich SF, Crites YM, Hedderson MM, Darbinian JA, Ferrara A. The risk of large for gestational age across increasing categories of pregnancy glycemia. Am J Obstet Gynecol. 2011;204(3):240.e1-6. 10.1016/j.ajog.2010.10.907 PMC3053062 21247550

[23] Zou G. A modified poisson regression approach to prospective studies with binary data. Am J Epidemiol. 2004;159(7):702–6. 15033648 10.1093/aje/kwh090

[24] Metzger BE, Lowe LP, Dyer AR, Trimble ER, Chaovarindr U, Coustan DR, et al Hyperglycemia and adverse pregnancy outcomes. N Engl J Med. 2008;358(19):1991–2002. 10.1056/NEJMoa0707943 18463375

[25] Lowe WL Jr., Scholtens DM, Lowe LP, Kuang A, Nodzenski M, Talbot O, et al Association of Gestational Diabetes With Maternal Disorders of Glucose Metabolism and Childhood Adiposity. JAMA. 2018;320(10):1005–16. 10.1001/jama.2018.11628 30208453 PMC6143108

[26] Thaware PK, McKenna S, Patterson CC, Hadden DR, Pettitt DJ, McCance DR. Untreated Mild Hyperglycemia During Pregnancy and Anthropometric Measures of Obesity in Offspring at Age 5–7 Years. Diabetes Care. 2015;38(9):1701–6. 10.2337/dc14-2797 26092862 PMC4542272

[27] Tam WH, Ma RCW, Ozaki R, Li AM, Chan MHM, Yuen LY, et al In Utero Exposure to Maternal Hyperglycemia Increases Childhood Cardiometabolic Risk in Offspring. Diabetes Care. 2017;40(5):679–86. 10.2337/dc16-2397 28279981 PMC5399651

[28] Wang J, Pan L, Liu E, Liu H, Liu J, Wang S, et al Gestational diabetes and offspring's growth from birth to 6 years old. Int J Obes (Lond). 2018. 10.1038/s41366-018-0193-z PMC6532057 30181654

[29] Landon MB, Rice MM, Varner MW, Casey BM, Reddy UM, Wapner RJ, et al Mild gestational diabetes mellitus and long-term child health. Diabetes Care. 2015;38(3):445–52. 10.2337/dc14-2159 25414152 PMC4338507

[30] Hedderson MM, Gunderson EP, Ferrara A. Gestational weight gain and risk of gestational diabetes mellitus. Obstet Gynecol. 2010;115(3):597–604. 10.1097/AOG.0b013e3181cfce4f 20177292 PMC3180899

[31] Sridhar SB, Darbinian J, Ehrlich SF, Markman MA, Gunderson EP, Ferrara A, et al Maternal gestational weight gain and offspring risk for childhood overweight or obesity. Am J Obstet Gynecol. 2014;211(3):259.e1-8. 10.1016/j.ajog.2014.02.030 PMC5084619 24735804

