# Supplementary material for: Diagnostic thresholds for pregnancy hyperglycemia, maternal weight status and the risk of childhood obesity in a diverse Northern California cohort using health care delivery system data
Source: PLoS One. 2019 May 10;14(5):e0216897. doi: 10.1371/journal.pone.0216897 (PMC6510476; doi:10.1371/journal.pone.0216897)
Supplement: S1 Table — * Adjusted for maternal age, race-ethnicity, and BMI category. OGTT: 100g, 3-hr oral glucose tolerance test, IADPSG: International Association of Diabetes in Pregnancy Study Groups, CC: Carpenter and Coustan, NDDG: National Diabetes Data Group. Note that the diagnostic criteria categories are not mutually exclusive, RR estimates obtained from separate models. (DOCX) [file pone.0216897.s001.docx]

**Supplement Table 1.** Risk Ratio estimates and 95% Confidence Intervals for associations of the Diagnostic Criteria for gestational diabetes with Childhood Obesity at 5-7 years of age, identified by the Centers for Disease Control and Prevention’s growth standards, among 46,396 women delivering at Kaiser Permanente Northern California in 1995-2004.

|  |  |  | **Childhood Obesity** | | |
| --- | --- | --- | --- | --- | --- |
|  |  |  |  | **Unadjusted** | **Adjusted**^*^ |
|  | **N women** |  | **n**  **cases of childhood obesity** | **RR (95% CI)** | **RR**^*^ **(95% CI)** |
| **Diagnostic Criteria for GDM** |  |  |  |  |  |
| Normal screening | 38,184 |  | 5,831 | Reference | Reference |
| Abnormal screening | 8,212 |  | 1,529 | 1.22 (1.16, 1.28) | 1.08 (1.03, 1.14) |
| Abnormal screening and 1+ abnormal OGTT values by IADPSG | 4,431 |  | 906 | 1.34 (1.26, 1.43) | 1.11 (1.04, 1.18) |
| Abnormal screening and 1+ abnormal OGTT value by CC | 4,392 |  | 897 | 1.34 (1.26, 1.42) | 1.12 (1.05, 1.19) |
| Abnormal screening and 2+ abnormal OGTT values by CC | 2,731 |  | 565 | 1.35 (1.25, 1.46) | 1.11 (1.03, 1.20) |
| Abnormal screening and 2+ abnormal OGTT values by NDDG | 1,825 |  | 398 | 1.43 (1.31, 1.56) | 1.17 (1.07, 1.27) |

^*^ Adjusted for maternal age, race-ethnicity, and BMI category

OGTT: 100g, 3-hr oral glucose tolerance test, IADPSG: International Association of Diabetes in Pregnancy Study Groups, CC: Carpenter and Coustan, NDDG: National Diabetes Data Group

Note that the diagnostic criteria categories are not mutually exclusive, RR estimates obtained from separate models
