# Supplementary material for: Diagnostic thresholds for pregnancy hyperglycemia, maternal weight status and the risk of childhood obesity in a diverse Northern California cohort using health care delivery system data
Source: PLoS One. 2019 May 10;14(5):e0216897. doi: 10.1371/journal.pone.0216897 (PMC6510476; doi:10.1371/journal.pone.0216897)
Supplement: S2 Table — * Adjusted for maternal age, race-ethnicity, and BMI category. † Meeting the International Association of Diabetes in Pregnancy Study Groups threshold. ‡ Meeting the Carpenter and Coustan threshold. § Meeting National Diabetes Data Group threshold ¶ Meeting the International Association of Diabetes in Pregnancy Study Groups/Carpenter and Coustan thresholds, which are identical for the 1-hour time point. IADPSG: International Association of Diabetes in Pregnancy Study Groups, CC: Carpenter and Coustan, NDDG: National Diabetes Data Group. Note that the glucose threshold categories are not mutually exclusive, RR estimates obtained from separate models. (DOCX) [file pone.0216897.s002.docx]

**Supplement Table 2.** Risk Ratio estimates and 95% Confidence Intervals for the associations of the Fasting, 1-hour and 2-hour Glucose Threshold Categories with Childhood Obesity at 5-7 years of age, identified by the Centers for Disease Control and Prevention’s growth standards, among 46,396 women delivering at Kaiser Permanente Northern California in 1995-2004.

|  |  |  | **Childhood Obesity** | | |
| --- | --- | --- | --- | --- | --- |
|  |  |  |  | **Unadjusted** | **Adjusted**^*^ |
|  | **N**  **women** |  | **n**  **cases of childhood obesity** | **RR (95% CI)** | **RR**^*^ **(95% CI)** |
| **Fasting Glucose Thresholds** |  |  |  |  |  |
| Normal screening | 38,184 |  | 5,831 | Reference | Reference |
| Abnormal screening | 8,212 |  | 1,529 | 1.22 (1.16, 1.28) | 1.08 (1.03, 1.14) |
| Abnormal screening and fasting glucose ≥92 mg/dl^†^ | 1,751 |  | 447 | 1.65 (1.52, 1.79) | 1.19 (1.09, 1.29) |
| Abnormal screening and fasting glucose ≥95 mg/dl^‡^ | 1,277 |  | 344 | 1.76 (1.61, 1.94) | 1.24 (1.13, 1.36) |
| Abnormal screening and fasting glucose ≥105 mg/dl^§^ | 439 |  | 146 | 2.18 (1.90, 2.49) | 1.41 (1.23, 1.61) |
| **1-hour Glucose Thresholds** |  |  |  |  |  |
| Normal screening | 38,184 |  | 5,831 | Reference | Reference |
| Abnormal screening | 8,212 |  | 1,529 | 1.22 (1.16, 1.28) | 1.08 (1.03, 1.14) |
| Abnormal screening, 1-hour glucose ≥180 mg/dl^¶^ | 3,044 |  | 637 | 1.37 (1.27, 1.47) | 1.12 (1.05, 1.21) |
| Abnormal screening, 1-hour glucose ≥190 mg/dl^§^ | 2,183 |  | 471 | 1.41 (1.30, 1.54) | 1.13 (1.04, 1.23) |
| **2-hour Glucose Thresholds** |  |  |  |  |  |
| Normal screening | 38,184 |  | 5,831 | Reference | Reference |
| Abnormal screening | 8,212 |  | 1,529 | 1.22 (1.16, 1.28) | 1.08 (1.03, 1.14) |
| Abnormal screening, 2-hour glucose ≥153 mg/dl^†^ | 3,290 |  | 659 | 1.29 (1.20, 1.38) | 1.09 (1.02, 1.17) |
| Abnormal screening, 2-hour glucose ≥155 mg/dl^‡^ | 3,134 |  | 632 | 1.32 (1.23, 1.42) | 1.11 (1.03, 1.20) |
| Abnormal screening, 2-hour glucose ≥165 mg/dl^§^ | 2,239 |  | 453 | 1.32 (1.22, 1.44) | 1.11 (1.02, 1.21) |

^*^ Adjusted for maternal age, race-ethnicity, and BMI category

^†^ Meeting the International Association of Diabetes in Pregnancy Study Groups threshold

^‡^ Meeting the Carpenter and Coustan threshold

^§^ Meeting National Diabetes Data Group threshold

^¶^ Meeting the International Association of Diabetes in Pregnancy Study Groups/Carpenter and Coustan thresholds, which are identical for the 1-hour time point

IADPSG: International Association of Diabetes in Pregnancy Study Groups, CC: Carpenter and Coustan, NDDG: National Diabetes Data Group

Note that the glucose threshold categories are not mutually exclusive, RR estimates obtained from separate models
