# Supplementary material for: Diagnostic thresholds for pregnancy hyperglycemia, maternal weight status and the risk of childhood obesity in a diverse Northern California cohort using health care delivery system data
Source: PLoS One. 2019 May 10;14(5):e0216897. doi: 10.1371/journal.pone.0216897 (PMC6510476; doi:10.1371/journal.pone.0216897)
Supplement: S3 Table — * Multivariable models include the respective pregnancy glycemia variable, maternal age and BMI category (<18.5 kg/m2 and 18.5–24.9 kg/m2). † Meeting the International Association of Diabetes in Pregnancy Study Groups threshold. ‡ Meeting the Carpenter and Coustan threshold. § Meeting National Diabetes Data Group threshold. ¶ Meeting the International Association of Diabetes in Pregnancy Study Groups/Carpenter and Coustan thresholds, which are identical for the 1-hour time point. OGTT: 100g, 3-hr oral glucose tolerance test, IADPSG: International Association of Diabetes in Pregnancy Study Groups, CC: Carpenter and Coustan, NDDG: National Diabetes Data Group, CC: Carpenter and Coustan, NDDG: National Diabetes Data Group, BMI: body mass index Note that glucose categories are not mutually exclusive, RR estimates obtained from separate models. (DOCX) [file pone.0216897.s003.docx]

**Supplement Table 3.** Risk Ratio estimates and 95% Confidence Intervals for the associations of the GDM Diagnostic Criteria and Glucose Threshold Categories with Childhood Obesity at 5-7 years of age, identified by International Obesity Task Force’s cut-offs, among underweight and normal weight women (n= 22,126), Kaiser Permanente Northern California, 1995-2011.

|  |  |  | **Childhood Obesity** | | |
| --- | --- | --- | --- | --- | --- |
|  |  |  |  | **Unadjusted** | **Adjusted**^*^ |
|  | **N women** |  | **n**  **cases of childhood obesity** | **RR (95% CI)** | **RR**^*^ **(95% CI)** |
| **Underweight and Normal Weight Women** |  |  |  |  |  |
| **Non-mutually Exclusive Categories based on the Diagnostic Criteria for GDM** |  |  |  |  |  |
| Normal screening | 18,939 |  | 876 | Reference | Reference |
| Abnormal screening | 3,187 |  | 187 | 1.27 (1.09, 1.48) | 1.34 (1.15, 1.57) |
| Abnormal screening and 1+ abnormal OGTT values by IADPSG | 1,449 |  | 84 | 1.25 (1.01, 1.56) | 1.34 (1.07, 1.66) |
| Abnormal screening and 1+ abnormal OGTT value by CC | 1,469 |  | 89 | 1.31 (1.06, 1.62) | 1.40 (1.13, 1.73) |
| Abnormal screening and 2+ abnormal OGTT values by CC | 863 |  | 49 | 1.23 (0.93, 1.62) | 1.33 (1.00, 1.76) |
| Abnormal screening and 2+ abnormal OGTT values by NDDG | 553 |  | 28 | 1.09 (0.76, 1.58) | 1.18 (0.82, 1.71) |
| **Non-mutually Exclusive Categories based on the Time Point Specific Thresholds** |  |  |  |  |  |
| **Fasting** |  |  |  |  |  |
| Normal screening | 18,939 |  | 876 | Reference | Reference |
| Abnormal screening | 3,187 |  | 187 | 1.27 (1.09, 1.48) | 1.34 (1.15, 1.57) |
| Abnormal screening and fasting glucose ≥92 mg/dl^†^ | 330 |  | 19 | 1.20 (0.77, 1.87) | 1.27 (0.82, 1.97) |
| Abnormal screening and fasting glucose ≥95 mg/dl^‡^ | 228 |  | 14 | 1.33 (0.80, 2.21) | 1.40 (0.84, 2.34) |
| Abnormal screening and fasting glucose ≥105 mg/dl^§^ | 64 |  | 6 | 2.03 (0.94, 4.35) | 2.20 (1.03, 4.73) |
| **1-hour** |  |  |  |  |  |
| Normal screening | 18,939 |  | 876 | Reference | Reference |
| Abnormal screening | 3,187 |  | 187 | 1.27 (1.09, 1.48) | 1.34 (1.15, 1.57) |
| Abnormal screening, 1-hour glucose ≥180 mg/dl^¶^ | 942 |  | 55 | 1.26 (0.97, 1.64) | 1.34 (1.03, 1.75) |
| Abnormal screening, 1-hour glucose ≥190 mg/dl^§^ | 620 |  | 36 | 1.26 (0.91, 1.73) | 1.33 (0.96, 1.84) |
| **2-hour** |  |  |  |  |  |
| Normal screening | 18,939 |  | 876 | Reference | Reference |
| Abnormal screening | 3,187 |  | 187 | 1.27 (1.09, 1.48) | 1.34 (1.15, 1.57) |
| Abnormal screening, 2-hour glucose ≥153 mg/dl^†^ | 1,121 |  | 56 | 1.04 (0.80, 1.35) | 1.11 (0.86, 1.45) |
| Abnormal screening, 2-hour glucose ≥155 mg/dl^‡^ | 1,065 |  | 53 | 1.08 (0.82, 1.41) | 1.16 (0.88, 1.52) |
| Abnormal screening, 2-hour glucose ≥165 mg/dl^§^ | 744 |  | 36 | 1.05 (0.76, 1.45) | 1.14 (0.82, 1.58) |

^*^ Multivariable models include the respective pregnancy glycemia variable, maternal age and BMI category (<18.5 kg/m^2^ and 18.5-24.9 kg/m^2^)

^†^ Meeting the International Association of Diabetes in Pregnancy Study Groups threshold

^‡^ Meeting the Carpenter and Coustan threshold

^§^ Meeting National Diabetes Data Group threshold

^¶^ Meeting the International Association of Diabetes in Pregnancy Study Groups/Carpenter and Coustan thresholds, which are identical for the 1-hour time point

OGTT: 100g, 3-hr oral glucose tolerance test, IADPSG: International Association of Diabetes in Pregnancy Study Groups, CC: Carpenter and Coustan, NDDG: National Diabetes Data Group, CC: Carpenter and Coustan, NDDG: National Diabetes Data Group, BMI: body mass index

Note that glucose categories are not mutually exclusive, RR estimates obtained from separate models
