# Supplementary material for: Diagnostic thresholds for pregnancy hyperglycemia, maternal weight status and the risk of childhood obesity in a diverse Northern California cohort using health care delivery system data
Source: PLoS One. 2019 May 10;14(5):e0216897. doi: 10.1371/journal.pone.0216897 (PMC6510476; doi:10.1371/journal.pone.0216897)
Supplement: S4 Table — * Multivariable models include the respective pregnancy glycemia variable, maternal age and BMI category (25.0–29.9 kg/m2 and ≥30.0 kg/m2) † Meeting the International Association of Diabetes in Pregnancy Study Groups threshold. ‡ Meeting the Carpenter and Coustan threshold. § Meeting National Diabetes Data Group threshold. ¶ Meeting the International Association of Diabetes in Pregnancy Study Groups/Carpenter and Coustan thresholds, which are identical for the 1-hour time point. OGTT: 100g, 3-hr oral glucose tolerance test, IADPSG: International Association of Diabetes in Pregnancy Study Groups, CC: Carpenter and Coustan, NDDG: National Diabetes Data Group, CC: Carpenter and Coustan, NDDG: National Diabetes Data Group, BMI: body mass index. Note that glucose categories are not mutually exclusive, RR estimates obtained from separate models. (DOCX) [file pone.0216897.s004.docx]

**Supplement Table 4.** Risk Ratio estimates and 95% Confidence Intervals for the associations of the GDM Diagnostic Criteria and Glucose Threshold Categories with Childhood Obesity at 5-7 years of age, identified by International Obesity Task Force’s cut-offs, among women with overweight or obesity (n= 24,270), Kaiser Permanente Northern California, 1995-2011.

|  |  |  | **Childhood Obesity** | | |
| --- | --- | --- | --- | --- | --- |
|  |  |  |  | **Unadjusted** | **Adjusted**^*^ |
|  | **N women** |  | **n**  **cases of childhood obesity** | **RR (95% CI)** | **RR**^*^ **(95% CI)** |
| **Women with Overweight or Obesity** |  |  |  |  |  |
| **Non-mutually Exclusive Categories based on the Diagnostic Criteria for GDM** |  |  |  |  |  |
| Normal screening | 19,245 |  | 2,954 | Reference | Reference |
| Abnormal screening | 5,025 |  | 883 | 1.14 (1.07, 1.23) | 1.11 (1.04, 1.19) |
| Abnormal screening and 1+ abnormal OGTT values by IADPSG | 2,982 |  | 570 | 1.25 (1.15, 1.35) | 1.19 (1.10, 1.29) |
| Abnormal screening and 1+ abnormal OGTT value by CC | 2,923 |  | 561 | 1.25 (1.15, 1.36) | 1.20 (1.10, 1.30) |
| Abnormal screening and 2+ abnormal OGTT values by CC | 1,868 |  | 368 | 1.28 (1.16, 1.41) | 1.23 (1.11, 1.35) |
| Abnormal screening and 2+ abnormal OGTT values by NDDG | 1,272 |  | 264 | 1.35 (1.21, 1.51) | 1.30 (1.16, 1.45) |
| **Non-mutually Exclusive Categories based on the Time Point Specific Thresholds** |  |  |  |  |  |
| **Fasting** |  |  |  |  |  |
| Normal screening | 19,245 |  | 2,954 | Reference | Reference |
| Abnormal screening | 5,025 |  | 883 | 1.14 (1.07, 1.23) | 1.11 (1.04, 1.19) |
| Abnormal screening and fasting glucose ≥92 mg/dl^†^ | 1,421 |  | 315 | 1.44 (1.30, 1.59) | 1.30 (1.17, 1.44) |
| Abnormal screening and fasting glucose ≥95 mg/dl^‡^ | 1,049 |  | 245 | 1.52 (1.36, 1.71) | 1.37 (1.22, 1.54) |
| Abnormal screening and fasting glucose ≥105 mg/dl^§^ | 375 |  | 112 | 1.95 (1.66, 2.28) | 1.66 (1.41, 1.95) |
| **1-hour** |  |  |  |  |  |
| Normal screening | 19,245 |  | 2,954 | Reference | Reference |
| Abnormal screening | 5,025 |  | 883 | 1.14 (1.07, 1.23) | 1.11 (1.04, 1.19) |
| Abnormal screening, 1-hour glucose ≥180 mg/dl^¶^ | 2,102 |  | 423 | 1.31 (1.20, 1.44) | 1.26 (1.15, 1.38) |
| Abnormal screening, 1-hour glucose ≥190 mg/dl^§^ | 1,563 |  | 317 | 1.32 (1.19, 1.47) | 1.26 (1.14, 1.40) |
| **2-hour** |  |  |  |  |  |
| Normal screening | 19,245 |  | 2,954 | Reference | Reference |
| Abnormal screening | 5,025 |  | 883 | 1.14 (1.07, 1.23) | 1.11 (1.04, 1.19) |
| Abnormal screening, 2-hour glucose ≥153 mg/dl^†^ | 2,169 |  | 422 | 1.26 (1.15, 1.38) | 1.21 (1.11, 1.33) |
| Abnormal screening, 2-hour glucose ≥155 mg/dl^‡^ | 2,069 |  | 409 | 1.29 (1.17, 1.41) | 1.24 (1.13, 1.36) |
| Abnormal screening, 2-hour glucose ≥165 mg/dl^§^ | 1,495 |  | 289 | 1.26 (1.13, 1.40) | 1.22 (1.10, 1.36) |

^*^ Multivariable models include the respective pregnancy glycemia variable, maternal age and BMI category (25.0-29.9 kg/m^2^ and ≥30.0 kg/m^2^)

^†^ Meeting the International Association of Diabetes in Pregnancy Study Groups threshold

^‡^ Meeting the Carpenter and Coustan threshold

^§^ Meeting National Diabetes Data Group threshold

^¶^ Meeting the International Association of Diabetes in Pregnancy Study Groups/Carpenter and Coustan thresholds, which are identical for the 1-hour time point

OGTT: 100g, 3-hr oral glucose tolerance test, IADPSG: International Association of Diabetes in Pregnancy Study Groups, CC: Carpenter and Coustan, NDDG: National Diabetes Data Group, CC: Carpenter and Coustan, NDDG: National Diabetes Data Group, BMI: body mass index

Note that glucose categories are not mutually exclusive, RR estimates obtained from separate models
