# Supplementary material for: Diagnostic thresholds for pregnancy hyperglycemia, maternal weight status and the risk of childhood obesity in a diverse Northern California cohort using health care delivery system data
Source: PLoS One. 2019 May 10;14(5):e0216897. doi: 10.1371/journal.pone.0216897 (PMC6510476; doi:10.1371/journal.pone.0216897)
Supplement: S5 Table — * Multivariable models include the respective pregnancy glycemia variable, maternal age and BMI category (<18.5 kg/m2, 18.5–24.9 kg/m2, 25.0–29.9 kg/m2, and ≥30.0 kg/m2). † Meeting the International Association of Diabetes in Pregnancy Study Groups threshold ‡ Meeting the Carpenter and Coustan threshold. § Meeting National Diabetes Data Group threshold. ¶ Meeting the International Association of Diabetes in Pregnancy Study Groups/Carpenter and Coustan thresholds, which are identical for the 1-hour time point. OGTT: 100g, 3-hr oral glucose tolerance test, IADPSG: International Association of Diabetes in Pregnancy Study Groups, CC: Carpenter and Coustan, NDDG: National Diabetes Data Group, CC: Carpenter and Coustan, NDDG: National Diabetes Data Group, BMI: body mass index. Note that glucose categories are not mutually exclusive, RR estimates obtained from separate models. (DOCX) [file pone.0216897.s005.docx]

**Supplement Table 5.** Risk Ratio estimates and 95% Confidence Intervals for the associations of the GDM Diagnostic Criteria and Glucose Threshold Categories with Childhood Obesity at 5-7 years of age, identified by International Obesity Task Force’s cut-offs, among White women (n= 17,254), Kaiser Permanente Northern California, 1995-2011.

|  |  |  | **Childhood Obesity** | | |
| --- | --- | --- | --- | --- | --- |
|  |  |  |  | **Unadjusted** | **Adjusted**^*^ |
|  | **N women** |  | **n**  **cases of childhood obesity** | **RR (95% CI)** | **RR**^*^ **(95% CI)** |
| **White Women** |  |  |  |  |  |
| **Non-mutually Exclusive Categories based on the Diagnostic Criteria for GDM** |  |  |  |  |  |
| Normal screening | 14,753 |  | 1,044 | Reference | Reference |
| Abnormal screening | 2,501 |  | 262 | 1.48 (1.30, 1.68) | 1.20 (1.05, 1.36) |
| Abnormal screening and 1+ abnormal OGTT values by IADPSG | 1,233 |  | 158 | 1.81 (1.55, 2.12) | 1.30 (1.12, 1.52) |
| Abnormal screening and 1+ abnormal OGTT value by CC | 1,206 |  | 155 | 1.82 (1.55, 2.13) | 1.32 (1.12, 1.54) |
| Abnormal screening and 2+ abnormal OGTT values by CC | 728 |  | 90 | 1.75 (1.43, 2.14) | 1.26 (1.03, 1.54) |
| Abnormal screening and 2+ abnormal OGTT values by NDDG | 462 |  | 62 | 1.90 (1.49, 2.41) | 1.31 (1.03, 1.66) |
| **Non-mutually Exclusive Categories based on the Time Point Specific Thresholds** |  |  |  |  |  |
| **Fasting** |  |  |  |  |  |
| Normal screening | 14,753 |  | 1,044 | Reference | Reference |
| Abnormal screening | 2,501 |  | 262 | 1.48 (1.30, 1.68) | 1.20 (1.05, 1.36) |
| Abnormal screening and fasting glucose ≥92 mg/dl^†^ | 491 |  | 88 | 2.47 (2.03, 3.00) | 1.50 (1.23, 1.83) |
| Abnormal screening and fasting glucose ≥95 mg/dl^‡^ | 347 |  | 66 | 2.69 (2.15, 3.37) | 1.60 (1.28, 2.01) |
| Abnormal screening and fasting glucose ≥105 mg/dl^§^ | 103 |  | 26 | 3.57 (2.55, 5.00) | 2.02 (1.44, 2.84) |
| **1-hour** |  |  |  |  |  |
| Normal screening | 14,753 |  | 1,044 | Reference | Reference |
| Abnormal screening | 2,501 |  | 262 | 1.48 (1.30, 1.68) | 1.20 (1.05, 1.36) |
| Abnormal screening, 1-hour glucose ≥180 mg/dl^¶^ | 806 |  | 110 | 1.93 (1.61, 2.32) | 1.35 (1.12, 1.62) |
| Abnormal screening, 1-hour glucose ≥190 mg/dl^§^ | 545 |  | 77 | 2.00 (1.61, 2.48) | 1.32 (1.06, 1.63) |
| **2-hour** |  |  |  |  |  |
| Normal screening | 14,753 |  | 1,044 | Reference | Reference |
| Abnormal screening | 2,501 |  | 262 | 1.48 (1.30, 1.68) | 1.20 (1.05, 1.36) |
| Abnormal screening, 2-hour glucose ≥153 mg/dl^†^ | 903 |  | 108 | 1.63 (1.36, 1.96) | 1.24 (1.03, 1.49) |
| Abnormal screening, 2-hour glucose ≥155 mg/dl^‡^ | 859 |  | 104 | 1.71 (1.42, 2.07) | 1.28 (1.06, 1.55) |
| Abnormal screening, 2-hour glucose ≥165 mg/dl^§^ | 591 |  | 69 | 1.65 (1.31, 2.08) | 1.22 (0.98, 1.53) |

^*^ Multivariable models include the respective pregnancy glycemia variable, maternal age and BMI category (<18.5 kg/m^2^, 18.5-24.9 kg/m^2^, 25.0-29.9 kg/m^2^, and ≥30.0 kg/m^2^)

^†^ Meeting the International Association of Diabetes in Pregnancy Study Groups threshold

^‡^ Meeting the Carpenter and Coustan threshold

^§^ Meeting National Diabetes Data Group threshold

^¶^ Meeting the International Association of Diabetes in Pregnancy Study Groups/Carpenter and Coustan thresholds, which are identical for the 1-hour time point

OGTT: 100g, 3-hr oral glucose tolerance test, IADPSG: International Association of Diabetes in Pregnancy Study Groups, CC: Carpenter and Coustan, NDDG: National Diabetes Data Group, CC: Carpenter and Coustan, NDDG: National Diabetes Data Group, BMI: body mass index

Note that glucose categories are not mutually exclusive, RR estimates obtained from separate models
