# Supplementary material for: Diagnostic thresholds for pregnancy hyperglycemia, maternal weight status and the risk of childhood obesity in a diverse Northern California cohort using health care delivery system data
Source: PLoS One. 2019 May 10;14(5):e0216897. doi: 10.1371/journal.pone.0216897 (PMC6510476; doi:10.1371/journal.pone.0216897)
Supplement: S6 Table — * Multivariable models include the respective pregnancy glycemia variable, maternal age and BMI category (<24.9 kg/m2, 25.0–29.9 kg/m2, and ≥30.0 kg/m2). † Meeting the International Association of Diabetes in Pregnancy Study Groups threshold. ‡ Meeting the Carpenter and Coustan threshold. § Meeting National Diabetes Data Group threshold ¶ Meeting the International Association of Diabetes in Pregnancy Study Groups/Carpenter and Coustan thresholds, which are identical for the 1-hour time point. OGTT: 100g, 3-hr oral glucose tolerance test, IADPSG: International Association of Diabetes in Pregnancy Study Groups, CC: Carpenter and Coustan, NDDG: National Diabetes Data Group, CC: Carpenter and Coustan, NDDG: National Diabetes Data Group, BMI: body mass index. Note that glucose categories are not mutually exclusive, RR estimates obtained from separate models. (DOCX) [file pone.0216897.s006.docx]

**Supplement Table 6.** Risk Ratio estimates and 95% Confidence Intervals for the associations of the GDM Diagnostic Criteria and Glucose Threshold Categories with Childhood Obesity at 5-7 years of age, identified by International Obesity Task Force’s cut-offs, among Hispanic women (n= 12,740), Kaiser Permanente Northern California, 1995-2011.

|  |  |  | **Childhood Obesity** | | |
| --- | --- | --- | --- | --- | --- |
|  |  |  |  | **Unadjusted** | **Adjusted**^*^ |
|  | **N women** |  | **n**  **cases of childhood obesity** | **RR (95% CI)** | **RR**^*^ **(95% CI)** |
| **Hispanic Women** |  |  |  |  |  |
| **Non-mutually Exclusive Categories based on the Diagnostic Criteria for GDM** |  |  |  |  |  |
| Normal screening | 10,381 |  | 1,573 | Reference | Reference |
| Abnormal screening | 2,359 |  | 437 | 1.22 (1.11, 1.35) | 1.07 (0.97, 1.18) |
| Abnormal screening and 1+ abnormal OGTT values by IADPSG | 1,253 |  | 261 | 1.37 (1.22, 1.55) | 1.10 (0.98, 1.24) |
| Abnormal screening and 1+ abnormal OGTT value by CC | 1,234 |  | 255 | 1.36 (1.21, 1.54) | 1.11 (0.99, 1.25) |
| Abnormal screening and 2+ abnormal OGTT values by CC | 767 |  | 170 | 1.46 (1.27, 1.68) | 1.16 (1.00, 1.33) |
| Abnormal screening and 2+ abnormal OGTT values by NDDG | 496 |  | 117 | 1.56 (1.32, 1.84) | 1.24 (1.05, 1.46) |
| **Non-mutually Exclusive Categories based on the Time Point Specific Thresholds** |  |  |  |  |  |
| **Fasting** |  |  |  |  |  |
| Normal screening | 10,381 |  | 1,573 | Reference | Reference |
| Abnormal screening | 2,359 |  | 437 | 1.22 (1.11, 1.35) | 1.07 (0.97, 1.18) |
| Abnormal screening and fasting glucose ≥92 mg/dl^†^ | 563 |  | 140 | 1.62 (1.39, 1.88) | 1.22 (1.05, 1.41) |
| Abnormal screening and fasting glucose ≥95 mg/dl^‡^ | 425 |  | 109 | 1.69 (1.43, 2.00) | 1.22 (1.03, 1.45) |
| Abnormal screening and fasting glucose ≥105 mg/dl^§^ | 153 |  | 48 | 2.07 (1.63, 2.63) | 1.41 (1.10, 1.79) |
| **1-hour** |  |  |  |  |  |
| Normal screening | 10,381 |  | 1,573 | Reference | Reference |
| Abnormal screening | 2,359 |  | 437 | 1.22 (1.11, 1.35) | 1.07 (0.97, 1.18) |
| Abnormal screening, 1-hour glucose ≥180 mg/dl^¶^ | 855 |  | 193 | 1.49 (1.31, 1.70) | 1.18 (1.03, 1.35) |
| Abnormal screening, 1-hour glucose ≥190 mg/dl^§^ | 603 |  | 142 | 1.55 (1.34, 1.81) | 1.22 (1.05, 1.42) |
| **2-hour** |  |  |  |  |  |
| Normal screening | 10,381 |  | 1,573 | Reference | Reference |
| Abnormal screening | 2,359 |  | 437 | 1.22 (1.11, 1.35) | 1.07 (0.97, 1.18) |
| Abnormal screening, 2-hour glucose ≥153 mg/dl^†^ | 913 |  | 192 | 1.37 (1.20, 1.56) | 1.13 (0.99, 1.29) |
| Abnormal screening, 2-hour glucose ≥155 mg/dl^‡^ | 862 |  | 186 | 1.42 (1.24, 1.63) | 1.16 (1.01, 1.32) |
| Abnormal screening, 2-hour glucose ≥165 mg/dl^§^ | 599 |  | 122 | 1.34 (1.14, 1.58) | 1.09 (0.92, 1.28) |

^*^ Multivariable models include the respective pregnancy glycemia variable, maternal age and BMI category (<24.9 kg/m^2^, 25.0-29.9 kg/m^2^, and ≥30.0 kg/m^2^)

^†^ Meeting the International Association of Diabetes in Pregnancy Study Groups threshold

^‡^ Meeting the Carpenter and Coustan threshold

^§^ Meeting National Diabetes Data Group threshold

^¶^ Meeting the International Association of Diabetes in Pregnancy Study Groups/Carpenter and Coustan thresholds, which are identical for the 1-hour time point

OGTT: 100g, 3-hr oral glucose tolerance test, IADPSG: International Association of Diabetes in Pregnancy Study Groups, CC: Carpenter and Coustan, NDDG: National Diabetes Data Group, CC: Carpenter and Coustan, NDDG: National Diabetes Data Group, BMI: body mass index

Note that glucose categories are not mutually exclusive, RR estimates obtained from separate models
