# Supplementary material for: Diagnostic thresholds for pregnancy hyperglycemia, maternal weight status and the risk of childhood obesity in a diverse Northern California cohort using health care delivery system data
Source: PLoS One. 2019 May 10;14(5):e0216897. doi: 10.1371/journal.pone.0216897 (PMC6510476; doi:10.1371/journal.pone.0216897)
Supplement: S7 Table — * Multivariable models include the respective pregnancy glycemia variable, maternal age and BMI category (<18.5 kg/m2, 18.5–24.9 kg/m2, 25.0–29.9 kg/m2, and ≥30.0 kg/m2). † Meeting the International Association of Diabetes in Pregnancy Study Groups threshold ‡ Meeting the Carpenter and Coustan threshold. § Meeting National Diabetes Data Group threshold. ¶ Meeting the International Association of Diabetes in Pregnancy Study Groups/Carpenter and Coustan thresholds, which are identical for the 1-hour time point. OGTT: 100g, 3-hr oral glucose tolerance test, IADPSG: International Association of Diabetes in Pregnancy Study Groups, CC: Carpenter and Coustan, NDDG: National Diabetes Data Group, CC: Carpenter and Coustan, NDDG: National Diabetes Data Group, BMI: body mass index. Note that glucose categories are not mutually exclusive, RR estimates obtained from separate models. (DOCX) [file pone.0216897.s007.docx]

**Supplement Table 7.** Risk Ratio estimates and 95% Confidence Intervals for the associations of the GDM Diagnostic Criteria and Glucose Threshold Categories with Childhood Obesity at 5-7 years of age, identified by International Obesity Task Force’s cut-offs, among Asian women (n= 10,823), Kaiser Permanente Northern California, 1995-2011.

|  |  |  | **Childhood Obesity** | | |
| --- | --- | --- | --- | --- | --- |
|  |  |  |  | **Unadjusted** | **Adjusted**^*^ |
|  | **N women** |  | **n**  **cases of childhood obesity** | **RR (95% CI)** | **RR**^*^ **(95% CI)** |
| **Asian Women** |  |  |  |  |  |
| **Non-mutually Exclusive Categories based on the Diagnostic Criteria for GDM** |  |  |  |  |  |
| Normal screening | 8,151 |  | 551 | Reference | Reference |
| Abnormal screening | 2,672 |  | 263 | 1.46 (1.27, 1.68) | 1.26 (1.09, 1.45) |
| Abnormal screening and 1+ abnormal OGTT values by IADPSG | 1,579 |  | 168 | 1.57 (1.34, 1.85) | 1.27 (1.08, 1.50) |
| Abnormal screening and 1+ abnormal OGTT value by CC | 1,590 |  | 175 | 1.63 (1.39, 1.91) | 1.32 (1.12, 1.56) |
| Abnormal screening and 2+ abnormal OGTT values by CC | 1,004 |  | 110 | 1.62 (1.34, 1.97) | 1.29 (1.06, 1.57) |
| Abnormal screening and 2+ abnormal OGTT values by NDDG | 697 |  | 77 | 1.63 (1.30, 2.05) | 1.26 (1.01, 1.58) |
| **Non-mutually Exclusive Categories based on the Time Point Specific Thresholds** |  |  |  |  |  |
| **Fasting** |  |  |  |  |  |
| Normal screening | 8,151 |  | 551 | Reference | Reference |
| Abnormal screening | 2,672 |  | 263 | 1.46 (1.27, 1.68) | 1.26 (1.09, 1.45) |
| Abnormal screening and fasting glucose ≥92 mg/dl^†^ | 504 |  | 63 | 1.72 (1.35, 2.19) | 1.20 (0.94, 1.53) |
| Abnormal screening and fasting glucose ≥95 mg/dl^‡^ | 355 |  | 48 | 2.00 (1.52, 2.63) | 1.38 (1.04, 1.83) |
| Abnormal screening and fasting glucose ≥105 mg/dl^§^ | 114 |  | 19 | 2.47 (1.62, 3.75) | 1.58 (1.03, 2.43) |
| **1-hour** |  |  |  |  |  |
| Normal screening | 8,151 |  | 551 | Reference | Reference |
| Abnormal screening | 2,672 |  | 263 | 1.46 (1.27, 1.68) | 1.26 (1.09, 1.45) |
| Abnormal screening, 1-hour glucose ≥180 mg/dl^¶^ | 1,138 |  | 124 | 1.61 (1.34, 1.94) | 1.26 (1.05, 1.51) |
| Abnormal screening, 1-hour glucose ≥190 mg/dl^§^ | 848 |  | 93 | 1.62 (1.32, 2.00) | 1.22 (0.99, 1.50) |
| **2-hour** |  |  |  |  |  |
| Normal screening | 8,151 |  | 551 | Reference | Reference |
| Abnormal screening | 2,672 |  | 263 | 1.46 (1.27, 1.68) | 1.26 (1.09, 1.45) |
| Abnormal screening, 2-hour glucose ≥153 mg/dl^†^ | 1,194 |  | 123 | 1.44 (1.20, 1.72) | 1.19 (0.99, 1.43) |
| Abnormal screening, 2-hour glucose ≥155 mg/dl^‡^ | 1,143 |  | 120 | 1.55 (1.29, 1.87) | 1.26 (1.04, 1.52) |
| Abnormal screening, 2-hour glucose ≥165 mg/dl^§^ | 848 |  | 95 | 1.66 (1.35, 2.04) | 1.33 (1.08, 1.64) |

^*^ Multivariable models include the respective pregnancy glycemia variable, maternal age and BMI category (<18.5 kg/m^2^, 18.5-24.9 kg/m^2^, 25.0-29.9 kg/m^2^, and ≥30.0 kg/m^2^)

^†^ Meeting the International Association of Diabetes in Pregnancy Study Groups threshold

^‡^ Meeting the Carpenter and Coustan threshold

^§^ Meeting National Diabetes Data Group threshold

^¶^ Meeting the International Association of Diabetes in Pregnancy Study Groups/Carpenter and Coustan thresholds, which are identical for the 1-hour time point

OGTT: 100g, 3-hr oral glucose tolerance test, IADPSG: International Association of Diabetes in Pregnancy Study Groups, CC: Carpenter and Coustan, NDDG: National Diabetes Data Group, CC: Carpenter and Coustan, NDDG: National Diabetes Data Group, BMI: body mass index

Note that glucose categories are not mutually exclusive, RR estimates obtained from separate models
