# Supplementary material for: Diagnostic thresholds for pregnancy hyperglycemia, maternal weight status and the risk of childhood obesity in a diverse Northern California cohort using health care delivery system data
Source: PLoS One. 2019 May 10;14(5):e0216897. doi: 10.1371/journal.pone.0216897 (PMC6510476; doi:10.1371/journal.pone.0216897)
Supplement: S8 Table — * Multivariable models include the respective pregnancy glycemia variable, maternal age and BMI category (<18.5 kg/m2, 18.5–24.9 kg/m2, 25.0–29.9 kg/m2, and ≥30.0 kg/m2). † Meeting the International Association of Diabetes in Pregnancy Study Groups threshold. ‡ Meeting the Carpenter and Coustan threshold. § Meeting National Diabetes Data Group threshold. ¶ Meeting the International Association of Diabetes in Pregnancy Study Groups/Carpenter and Coustan thresholds, which are identical for the 1-hour time point. OGTT: 100g, 3-hr oral glucose tolerance test, IADPSG: International Association of Diabetes in Pregnancy Study Groups, CC: Carpenter and Coustan, NDDG: National Diabetes Data Group, CC: Carpenter and Coustan, NDDG: National Diabetes Data Group, BMI: body mass index. Note that glucose categories are not mutually exclusive, RR estimates obtained from separate models. (DOCX) [file pone.0216897.s008.docx]

**Supplement Table 8.** Risk Ratio estimates and 95% Confidence Intervals for the associations of the GDM Diagnostic Criteria and Glucose Threshold Categories with Childhood Obesity at 5-7 years of age, identified by International Obesity Task Force’s cut-offs, among African American women (n= 4,051), Kaiser Permanente Northern California, 1995-2011.

|  |  |  | **Childhood Obesity** | | |
| --- | --- | --- | --- | --- | --- |
|  |  |  |  | **Unadjusted** | **Adjusted**^*^ |
|  | **N women** |  | **n**  **cases of childhood obesity** | **RR (95% CI)** | **RR**^*^ **(95% CI)** |
| **African American Women** |  |  |  |  |  |
| **Non-mutually Exclusive Categories based on the Diagnostic Criteria for GDM** |  |  |  |  |  |
| Normal screening | 3,634 |  | 515 | Reference | Reference |
| Abnormal screening | 417 |  | 75 | 1.27 (1.02, 1.58) | 1.03 (0.82, 1.28) |
| Abnormal screening and 1+ abnormal OGTT values by IADPSG | 227 |  | 47 | 1.46 (1.12, 1.91) | 1.08 (0.83, 1.41) |
| Abnormal screening and 1+ abnormal OGTT value by CC | 226 |  | 45 | 1.41 (1.07, 1.85) | 1.06 (0.81, 1.39) |
| Abnormal screening and 2+ abnormal OGTT values by CC | 155 |  | 35 | 1.59 (1.18, 2.16) | 1.18 (0.88, 1.59) |
| Abnormal screening and 2+ abnormal OGTT values by NDDG | 117 |  | 27 | 1.63 (1.16, 2.29) | 1.24 (0.89, 1.73) |
| **Non-mutually Exclusive Categories based on the Time Point Specific Thresholds** |  |  |  |  |  |
| **Fasting** |  |  |  |  |  |
| Normal screening | 3,634 |  | 515 | Reference | Reference |
| Abnormal screening | 417 |  | 75 | 1.27 (1.02, 1.58) | 1.03 (0.82, 1.28) |
| Abnormal screening and fasting glucose ≥92 mg/dl^†^ | 123 |  | 32 | 1.83 (1.35, 2.49) | 1.26 (0.92, 1.72) |
| Abnormal screening and fasting glucose ≥95 mg/dl^‡^ | 101 |  | 26 | 1.82 (1.29, 2.55) | 1.22 (0.87, 1.73) |
| Abnormal screening and fasting glucose ≥105 mg/dl^§^ | 46 |  | 16 | 2.45 (1.64, 3.68) | 1.56 (1.02, 2.38) |
| **1-hour** |  |  |  |  |  |
| Normal screening | 3,634 |  | 515 | Reference | Reference |
| Abnormal screening | 417 |  | 75 | 1.27 (1.02, 1.58) | 1.03 (0.82, 1.28) |
| Abnormal screening, 1-hour glucose ≥180 mg/dl^¶^ | 152 |  | 34 | 1.58 (1.16, 2.15) | 1.18 (0.87, 1.60) |
| Abnormal screening, 1-hour glucose ≥190 mg/dl^§^ | 118 |  | 29 | 1.73 (1.25, 2.40) | 1.28 (0.93, 1.77) |
| **2-hour** |  |  |  |  |  |
| Normal screening | 3,634 |  | 515 | Reference | Reference |
| Abnormal screening | 417 |  | 75 | 1.27 (1.02, 1.58) | 1.03 (0.82, 1.28) |
| Abnormal screening, 2-hour glucose ≥153 mg/dl^†^ | 187 |  | 42 | 1.58 (1.20, 2.09) | 1.20 (0.91, 1.57) |
| Abnormal screening, 2-hour glucose ≥155 mg/dl^‡^ | 182 |  | 40 | 1.55 (1.17, 2.06) | 1.17 (0.88, 1.54) |
| Abnormal screening, 2-hour glucose ≥165 mg/dl^§^ | 137 |  | 28 | 1.44 (1.03, 2.03) | 1.10 (0.79, 1.54) |

^*^ Multivariable models include the respective pregnancy glycemia variable, maternal age and BMI category (<18.5 kg/m^2^, 18.5-24.9 kg/m^2^, 25.0-29.9 kg/m^2^, and ≥30.0 kg/m^2^)

^†^ Meeting the International Association of Diabetes in Pregnancy Study Groups threshold

^‡^ Meeting the Carpenter and Coustan threshold

^§^ Meeting National Diabetes Data Group threshold

^¶^ Meeting the International Association of Diabetes in Pregnancy Study Groups/Carpenter and Coustan thresholds, which are identical for the 1-hour time point

OGTT: 100g, 3-hr oral glucose tolerance test, IADPSG: International Association of Diabetes in Pregnancy Study Groups, CC: Carpenter and Coustan, NDDG: National Diabetes Data Group, CC: Carpenter and Coustan, NDDG: National Diabetes Data Group, BMI: body mass index

Note that glucose categories are not mutually exclusive, RR estimates obtained from separate models
